# Focused Ultrasound-Modulated Glomerular Ultrafiltration Assessed by Functional Changes in Renal Arteries

**DOI:** 10.1371/journal.pone.0054034

**Published:** 2013-01-10

**Authors:** Feng-Yi Yang, Wei-Hsiu Chiu

**Affiliations:** Department of Biomedical Imaging and Radiological Sciences, School of Biomedical Science and Engineering, National Yang-Ming University, Taipei, Taiwan; National Cancer Institute, United States of America

## Abstract

This study demonstrates the feasibility of using focused ultrasound (FUS) to modulate glomerular ultrafiltration by renal artery sonication and determine if protein-creatinine ratios are estimated through vascular parameters. All animal experiments were approved by our Animal Care and Use Committee. The renal arteries of Sprague-Dawley rats were surgically exposed and sonicated at various acoustic power levels using a FUS transducer with a resonant frequency of 1 MHz. The mean peak systolic velocity (PSV) of the blood flow was measured by Doppler ultrasound imaging. Urinary protein-creatinine ratios were calculated during the experiments. Histological examination of renal arteries and whole kidneys was performed. The PSV, pulsatility index, and resistance index of blood flow significantly increased in the arteries after FUS sonication without microbubbles (*p*<0.05). The change in normalized protein-creatinine ratios significantly increased with increasing acoustic power, but such was not observed when microbubbles were administered. Furthermore, no histological changes were observed in the hematoxylin- and eosin-stained sections. Glomerular ultrafiltration is regulated temporarily by renal artery sonication without microbubbles. Monitoring vascular parameters are useful in estimating the normalized change in protein-creatinine ratios.

## Introduction

Focused ultrasound (FUS) generates mechanical effects, such as cavitation, radiation, and microjetting, which increase capillary permeability and produce transient nanopores in cell membranes [1], [2]. With microbubbles, FUS has evolved as a promising method for site-specific drug and gene delivery. In addition, FUS is effective in creating hemostasis of blood vessels and occluding blood flow in animal experiments [3], [4]. Recent studies have indicated that the magnitude of biological effects is dependent on acoustic pressure and the concentration of microbubbles [5], [6], [7].

The use of FUS with microbubbles has been proven to disrupt the blood–brain barrier (BBB) locally and provide a noninvasive tool to enhance the delivery of chemotherapeutic agents to the brain for treating brain tumors [8], [9], [10], [11]. The size selectivity of the glomerular filtration barrier (GFB) is also modulated with the simultaneous application of FUS and microbubbles to the kidneys [12]. Both BBB and GFB are vascular structures that act as barriers between blood and the surrounding tissues. The glomerular basement membrane (GBM) and its associated cells are critical elements in the glomerular ultrafiltration process. Through its physical properties, such as thickness and charge, GBM is altered dynamically. One major physiological property of GFB is its ability to restrict the flow of plasma proteins, such as albumin, into the urinary space [13].

In addition to the use of contrast-specific medical imaging tools to detect FUS- enhanced drug delivery [14], [15], [16], color Doppler imaging has been used to measure FUS-induced blood flow variation. Functional changes in arteries subjected to FUS vary with acoustic power, and treatments do not produce histological changes in vessel walls [17], [18], [19]. Monitoring peak systolic velocity (PSV) and pulsatility index (PI) are useful in the real-time evaluation of the concentration of drugs delivered to downstream sites during sonication. However, the relationship between the efficiency of drug delivery and FUS-induced vascular changes is still unknown. This study aims to test whether glomerular ultrafiltration is modulated by FUS exposure at the upstream renal artery of the glomerulus. Furthermore, functional changes may serve as indications for evaluating the protein-creatinine ratio in urine after FUS exposure.

## Results

**Figure 1:**
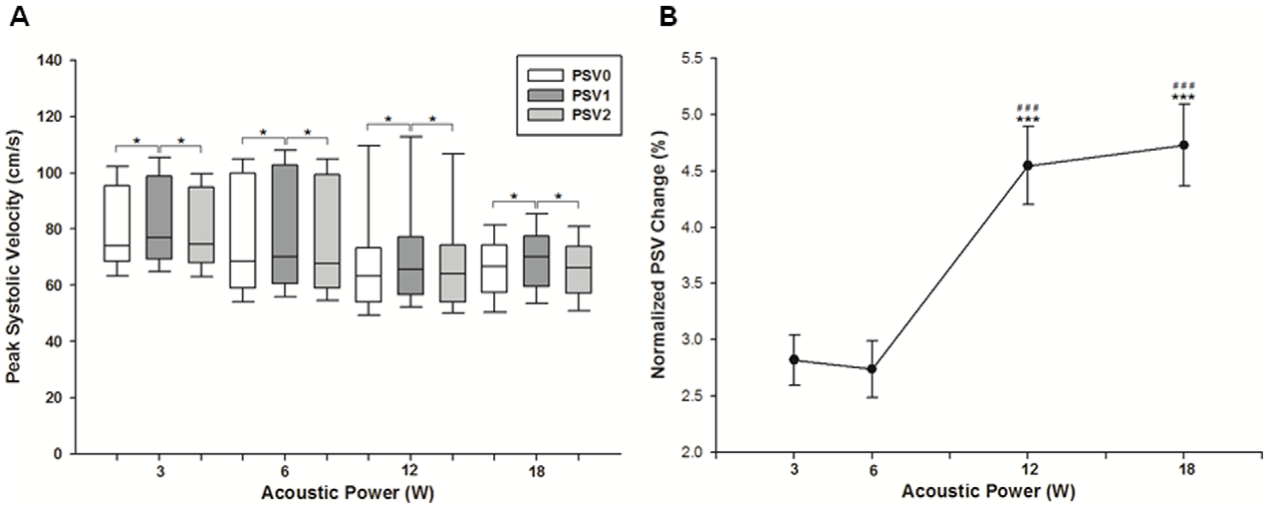
Changes in peak systolic velocity (PSV) of blood flow in the renal arteries of rats during FUS exposure. (A) The boxes extend from the 25^th^ to the 75^th^ percentile, with horizontal lines indicating the median. Bar lines indicate the 10^th^ and 90^th^ percentiles (* *p*<.05). (B) Normalized change in PSV (mean ± SEM) of blood flow in the renal arteries of rats in response to various acoustic power levels immediately before and after FUS exposure. The normalized change in PSV was significantly greater in the high-power group (12 or 18 W) than in the low-power group (3 or 6 W) (mean ± SEM; standard *t*-test; ***, ### *p*<.001).

**Figure 2:**
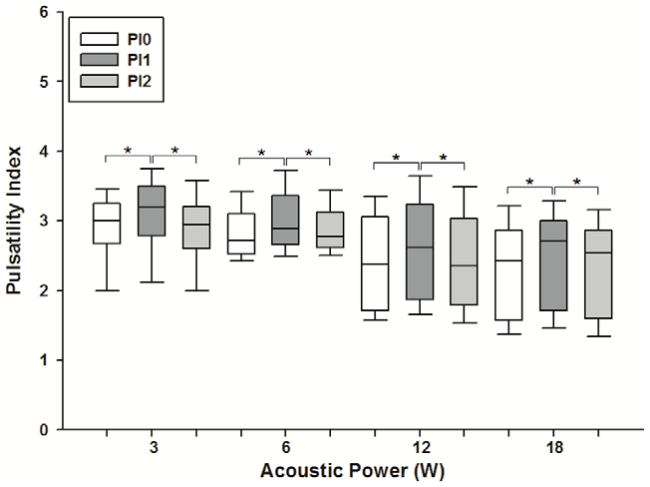
Changes in pulsatility index (PI) of blood flow in the renal arteries of rats immediately before and after FUS exposure. Significant changes in PI were observed at every acoustic power level. The boxes extend from the 25^th^ to the 75^th^ percentile, with horizontal lines indicating the median. Bar lines indicate the 10^th^ and 90^th^ percentiles (* *p*<.05).

**Figure 3:**
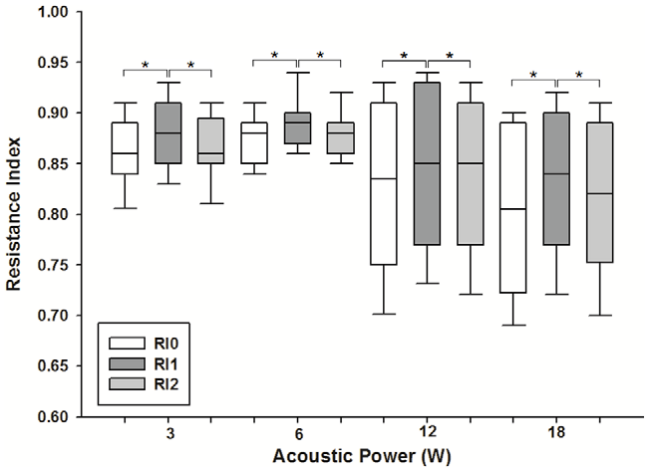
Changes in resistance index (RI) of blood flow in the renal arteries of rats immediately before and after FUS exposure. Significant changes in RI were observed at every acoustic power level. The boxes extend from the 25^th^ to the 75^th^ percentile, with horizontal lines indicating the median. Bar lines indicate the 10^th^ and 90^th^ percentiles (* *p*<.05).

Ultrasonographic measurements show that the mean inner diameter of the renal arteries was 1.12±0.06 mm. Blood flow velocity within the renal arteries was measured by color Doppler imaging. The average PSV taken before pulsed FUS exposure was 73.15±16.66 cm/s. The average PI and resistance index (RI) calculated from PSV were 2.62±0.60 and 0.84±0.06 cm/s, respectively. The median and 10^th^ and 90^th^ percentile PSV taken immediately after pulsed FUS exposure at 3, 6, 12, and 18 W (n = 105 for each group) were (77.0, 64.73, and 105.46 cm/s), (70.0, 55.95, and 108.11 cm/s), (65.65, 52.28, and 112.77 cm/s), and (70.2, 53.62, and 85.39 cm/s), respectively (Fig. 1A). The normalized change in PSV ((PSV_1_ – PSV_0_)/PSV_0_) at sonicated sites is shown in Fig. 1B. The amount of normalized change in PSV increased with increasing acoustic power of pulsed FUS, becoming significantly greater after sonication at 12 or 18 W than at 3 or 6 W (*p*<0.001). Similarly, statistically significant differences were found for PI and RI taken at sonicated sites immediately after sonication compared with those taken before sonication at four acoustic power levels (Figs. 2 and 3). However, significant differences for PSV, PI, and RI were not observed for the second values measured after sonication (Figs. 1A, 2, and 3).

**Figure 4:**
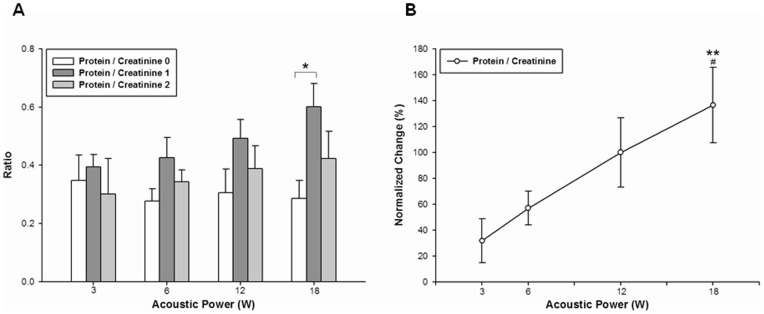
Protein-creatinine ratios before and after FUS treatment. (A) At the highest acoustic power of 18 W, the highest protein-creatinine ratio after FUS treatment was significantly different from the pretreatment value. (B) Graph showing the normalized change in urinary protein-creatinine ratios as a function of acoustic power. The normalized change for rats with arteries sonicated at the lower power levels of 3 and 6 W was significantly different from that for rats with arteries sonicated at the highest power level of 18 W (mean ± SEM; standard *t*-test; *, # *p*<.05; ** *p*<.01; n = 7 rats for each group).

**Figure 5:**
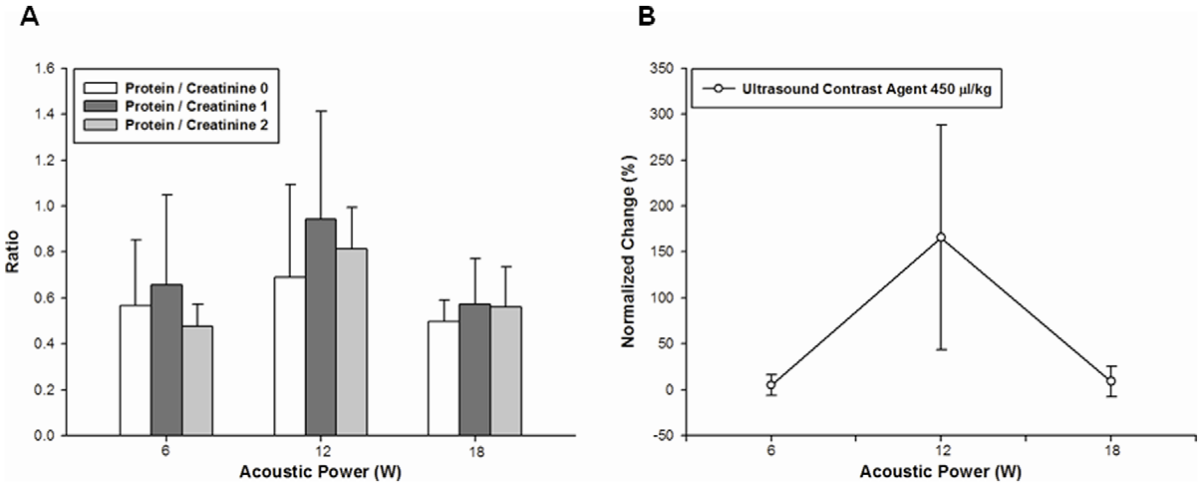
Protein-creatinine ratios before and after FUS treatment with microbubbles. (A) There are three acoustic powers at a dose of 450 μL/kg. (B) Graph showing the normalized change in urinary protein-creatinine ratios at different acoustic power levels with microbubbles at a dose of 450 μL/kg. No significant difference was found at various acoustic power levels (mean ± SEM; n = 3 rats for each group).

Figure 4A shows FUS treatment results at elevated protein-creatinine ratios within 1 h after sonication at all acoustic power levels. At the highest acoustic power of 18 W, enhancement ratios were higher compared to those prior to sonication. The elevated protein-creatinine ratios tended to return to their pre-sonication values. Immediately after sonication, the normalized change in protein-creatinine ratios increased as acoustic power was increased from 3 to 18 W (Fig. 4B). However, no significant differences were found after sonication in the presence of microbubbles for protein-creatinine ratios and their normalized change (Figs. 5A and 5B).

The protein-creatinine ratio is related to acoustic power, vessel diameter (VD), and functional changes. Multiple regression analysis revealed how the protein-creatinine ratio is related to acoustic power, VD, PSV, and PI, quantified as protein-creatinine ratio  = .046× acoustic power –.808× VD +13.993× normalized PSV +1124.138× normalized PI^3^ –400.184× normalized PI^2^ +38.896× normalized PI –.864 (R^2^  = 0.833, *p*<.001).

**Figure 6:**
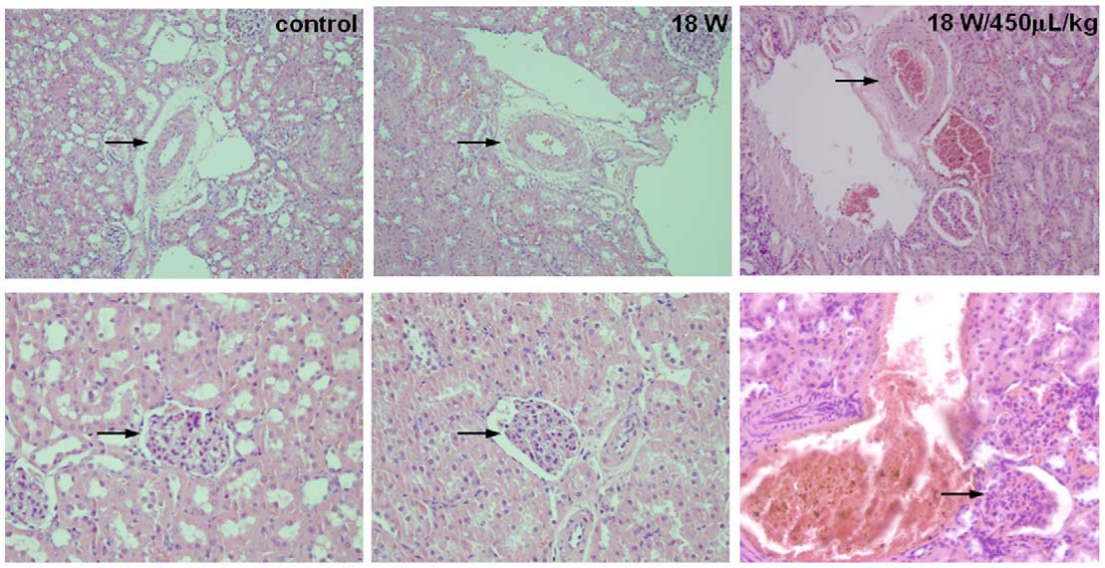
Microphotographs of hematoxylin- and eosin-stained sections of a renal artery (*top row, arrow*) and glomerulus (*bottom row, arrow*) after renal artery FUS treatment at the highest acoustic power level with and without microbubbles. No histological changes were found (original magnification ×200).

Hematoxylin- and eosin-stained sections of the renal arteries and glomeruli were observed and photographed with a light microscope immediately after sonication at the highest acoustic power with (Fig. 6, *right column*) and without microbubbles (Fig. 6, *middle column*). A histological study of the specimens resected immediately after pulsed FUS exposure showed no anatomic damage to the tissues as compared with the control tissues (Fig. 6, *left column*). Macroscopic examination of the sonicated sites showed no changes in the color of surrounding tissues or vessel walls in any of the experimental groups.

## Discussion

This study demonstrated that renal artery sonication with FUS affects the barrier function of the downstream glomerulus. Additionally, vascular changes induced by FUS may be useful in predicting protein-creatinine ratios after sonication. Our previous studies have shown that functional changes in vessels subjected to pulsed FUS vary with acoustic power and that treatments do not induce histological changes in vessel walls [17], [18], [19], [20]. Current results suggest the possibility of using an integrated pulsed FUS and ultrasound imaging system in the real-time monitoring of drug dose delivery.

Statistically significant differences were observed for PSV, PI, and RI before and immediately after pulsed FUS exposure (Figs. 1A, 2, and 3). However, the differences were clearly diminished 2 h after sonication, indicating that the vascular changes are transient. Based on previously published data, these results are expected. Gosling PI was originally designed to measure vascular resistance, and the above relationship has been proven in the brachial artery of normal humans [21], [22]. Meanwhile, RI has been suggested to indicate the vascular resistance of the peripheral part as seen from the measuring point. Hence, the increased PI or RI calculated in this study presumably represents enhanced vascular resistance around the sonicated renal arteries. Sonication with FUS possibly triggers a physiological response from the renal artery, which includes temporary vasoconstrictor stimulation and/or vasospasm. The permeability of the glomerular barrier may be enhanced because these effects transiently produce the increase of blood velocity, disturbed flow, and microstreaming in the downstream near the glomerulus.

Protein-creatinine ratios significantly increased within the first hour following pulsed FUS sonication but reached normal values within the second hour (Fig. 4A), suggesting that the increased glomerulus permeability caused by FUS exposure is temporary. In addition, significant differences were found at the highest acoustic power. The normalized change in protein-creatinine ratios increased as a function of acoustic power (Fig. 4B). Based on multiple regression analysis, monitoring the normalized change in PSV and PI may be useful in the real-time evaluation of drug dose delivery during sonication. Thus, the most appropriate FUS parameter for optimizing glomerular barrier disruption must be determined in further investigations. Interestingly, inconsistent with findings on urine produced by sonicated kidneys in a previous work [12], significant differences in protein-creatinine ratios were not observed after FUS exposure in the presence of ultrasound contrast agent (UCA) (Fig. 5). In the current study, glomerular permeability was probably modulated by FUS exposure at the renal arteries rather than at the kidneys. Glomerular permeability is enhanced by interactions between FUS and microbubbles at sonicated kidneys, but this effect was not observed when FUS with microbubbles was applied to renal arteries. A recent study has described arteriolar vasoconstriction on exposure to pulsed FUS with UCA and further showed that this disrupts blood flow [23]. Microbubbles, being compressible, alternately contract and expand in the ultrasound field, suggesting that bubble oscillation may reduce local blood flow. Moreover, microbubbles seem to produce bubble activity in the downstream site of sonicated vessels.

The most harmful event that is induced by FUS sonication is inertial cavitation, which may result in hemorrhage and apoptosis. No histological changes in the vessels or glomeruli were observed at the highest acoustic power with or without microbubbles (Fig. 6). Our results reveal that this acoustic power alone increases glomerular permeability by renal artery sonication without causing tissue damage, making it a potential powerful tool in the noninvasive treatment of renal diseases and in investigating the glomerular barrier function. In the cerebral vasculature, BBB disruption is caused by FUS exposure in the presence of UCA. Our experimental data yield valuable insights into improving the FUS-induced permeability of BBB and other tumor vessels for chemotherapy. Drug delivery to tumor sites may be improved by afferent artery sonication with FUS in the absence of microbubbles.

In conclusion, this study demonstrated that renal artery FUS exposure in the absence of microbubbles enhances, temporarily, the permeability of the glomerular barrier. Moreover, the normalized change in protein-creatinine ratios in urine from rats subjected to FUS is proportional to changes in acoustic power. The combination of FUS and ultrasound imaging is used in the real-time imaging and monitoring of drug delivery during sonication.

## Materials and Methods

### Pulsed FUS System

**Figure 7:**
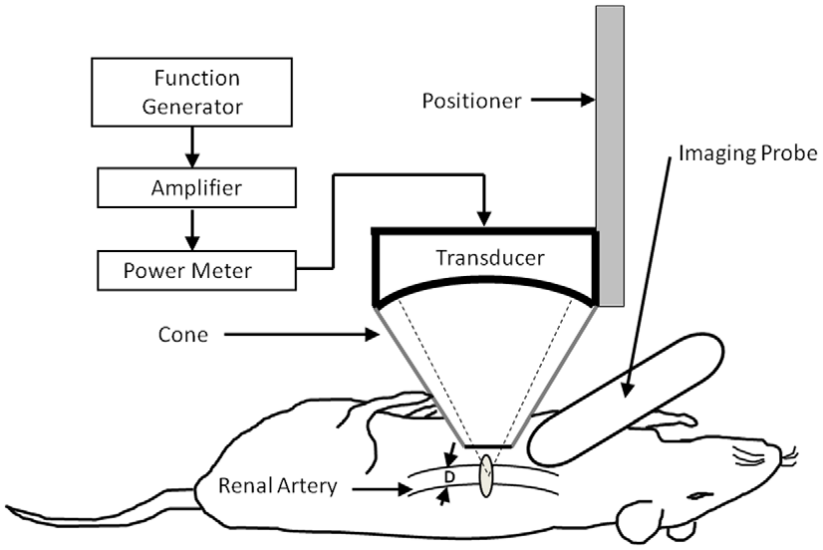
Schematic diagram of the experimental setup. The renal artery was exteriorized and sonicated with focused ultrasound (FUS). The diameter (D) of the renal artery was measured by ultrasound imaging.

Figure 7 shows the experimental setup. Pulsed sonications were performed using a single-element FUS transducer (H101MR, Sonic Concepts, Bothell, WA, USA) with an aperture diameter of 64 mm, curvature radius of 62.64 mm, and resonant frequency of 1.0 MHz. A removable cone was mounted on the transducer, filled with degassed water, and sealed with a polyurethane membrane. The center of the focal spot was approximately 5 mm below the cone tip. The transducer was attached to a stereotaxic apparatus (Stoelting, Wood Dale, IL, USA) to allow 3-D positioning. A function generator (33220A, Agilent Inc., Palo Alto, USA) was connected to a power amplifier (500–009, Advanced Surgical Systems, Tucson, AZ) to amplify FUS excitation signals. Signals were sent through a custom-built electrical matching network (matched to a 50 Ω load) to the FUS transducer. A power meter/sensor module (Bird 4421, Ohio, USA) was used to measure input electrical power. The focal zone of the therapeutic transducer was in the shape of an elongated ellipsoid, with a radial diameter (−6 dB) of 1.5 mm and an axial length (−6 dB) of 8 mm. The transducer-driving system is similar to that used in our previous work [24].

### Animal Preparation and Sonication

A total of 37 male Sprague-Dawley rats weighing 330–380 g were kept anesthetized intraperitoneally with chloral hydrate (400 mg/kg) throughout the experiment. All procedures were performed according to guidelines and approved by the Animal Care and Use Committee of the National Yang-Ming University. The renal arteries of anesthetized rats were surgically exposed for easy targeting and sonicated with pulsed FUS at a duty cycle of 5% and pulse repetition frequency of 1 Hz. Sonication lasted for 15 s for each rat. For the first experimental set, renal arteries were sonicated in the absence of microbubbles. We used 3, 6, 12, and 18 W acoustic powers, corresponding to the spatial average intensity of 116, 232, 464, and 696 W/cm^2^, respectively. For the second experimental set, renal arteries were sonicated with 6, 12, and 18 W acoustic powers in the presence of microbubbles at a dose of 450 μL/kg. An UCA (SonoVue, Bracco International, Amsterdam, The Netherlands), containing phospholipid-coated microbubbles (mean diameter of 2.5 μm) at a concentration of 1–5×10^8^ bubbles/ml, was injected into the tail vein of the rats about 20 s before each sonication.

### Calculation of Arterial Flow Indices

**Figure 8:**
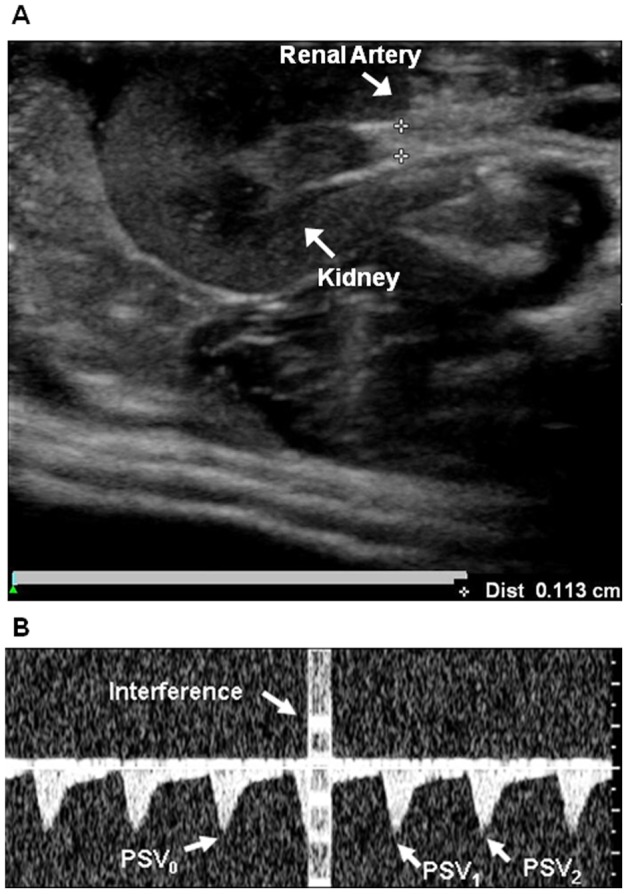
Measurement by ultrasound image. (A) B-mode ultrasound images of the renal artery and kidney of a rat. The inner diameter of the renal artery is about 1.1 mm. (B) Doppler spectral waveforms of blood flow in the renal artery of a rat during pulsed FUS exposure; interference caused by sonication (*bright column*) and the waveforms of peak systolic velocity (PSV) immediately before and after sonication (*indicated by arrows*).

An ultrasound system (iU22, Philips, Bothell, WA) was used to measure mean blood flow velocity during the cardiac cycle (MV), PSV, and diastolic velocity (DV) of blood flow. A linear array transducer (L15-7io, Philips) that could operate over a broad frequency range (7–15 MHz) was selected for its small footprint. The inner diameter of renal arteries was measured by B-mode imaging (Fig. 8A). Color Doppler imaging was used to target and monitor pulsed FUS beams at sonicated arterial sites. The angle between the vessel and Doppler beam was less than 60°. To monitor the effect of sonication on blood flow, blood velocity was measured immediately before and after exposure to spectral waveform in every case. In Fig. 8B, PSV_0_ is taken before sonication, whereas PSV_1_ and PSV_2_ are the first and second values, respectively, taken immediately after each pulsed FUS exposure. The formulae PI  =  (PSV – minimum DV)/MV and RI  =  (PSV – minimum DV)/PSV were used to calculate PI and RI, respectively. Meanwhile, PI_n_ and RI_n_ were calculated from PSV_n_ (n = 0, 1, and 2). Fifteen PSV measurements were taken at sonicated sites for each acoustic power and later analyzed in this study. The results are expressed as means ± SD. Differences were analyzed using the Wilcoxon signed-rank test. Statistical significance was defined as *p*≤.05.

### Urine Sample Collection and Urinalysis

Before and 1 h and 2 h after sonication, urine was obtained from the bladder of anesthetized rats using a catheter. Urinary protein and creatinine concentrations were measured using routine laboratory methods and the AUTION MAX AX-4030 (Arkray Inc., Tokyo, Japan) analyzer. Protein-creatinine ratios were computed for all measurements.

### Histological Observations

Four rats (exposed to FUS with or without microbubbles) from the treatment group and two untreated control rats were sacrificed for histology. The rats were perfused with saline and 10% neutral buffered formalin. The sonicated renal arteries were dissected, embedded in paraffin, and serially sectioned into 6 μm thick slices. The slices were stained with hematoxylin and eosin for general cellular structure visualization. Histological evaluation was conducted with a light microscope (BX61, Olympus).
